# Geranylgeranoic acid, a bioactive and endogenous fatty acid in mammals: a review

**DOI:** 10.1016/j.jlr.2023.100396

**Published:** 2023-05-27

**Authors:** Yoshihiro Shidoji

**Affiliations:** University of Nagasaki, Nagayo, Nagasaki, Japan

**Keywords:** caspase 4, ER stress-induced unfolded protein response, isoprenoids, pyroptosis, TLR4, UPR^ER^

## Abstract

Geranylgeranoic acid (GGA) was first reported in 1983 as one of the mevalonic acid metabolites, but its biological significance was not studied for a long time. Our research on the antitumor effects of retinoids led us to GGA, one of the acyclic retinoids that induce cell death in human hepatoma-derived cell lines. We were able to demonstrate the presence of endogenous GGA in various tissues of male rats, including the liver, testis, and cerebrum, by LC-MS/MS. Furthermore, the biosynthesis of GGA from mevalonic acid in mammals including humans was confirmed by isotopomer spectral analysis using ^13^C-labeled mevalonolactone and cultured hepatoma cells, and the involvement of hepatic monoamine oxidase B in the biosynthesis of GGA was also demonstrated. The biological activity of GGA was analyzed from the retinoid (differentiation induction) and nonretinoid (cell death induction) aspects, and in particular, the nonretinoid mechanism by which GGA induces cell death in hepatoma cells was found to involve pyroptosis via ER stress responses initiated by TLR4 signaling. In addition to these effects of GGA, we also describe the in vivo effects of GGA on reproduction. In this review, based mainly on our published papers, we have shown that hepatic monoamine oxidase B is involved in the biosynthesis of GGA and that GGA induces cell death in human hepatoma-derived cell lines by noncanonical pyroptosis, one of the mechanisms of sterile inflammatory cell death.

## Graphical abstract

**Figure:**
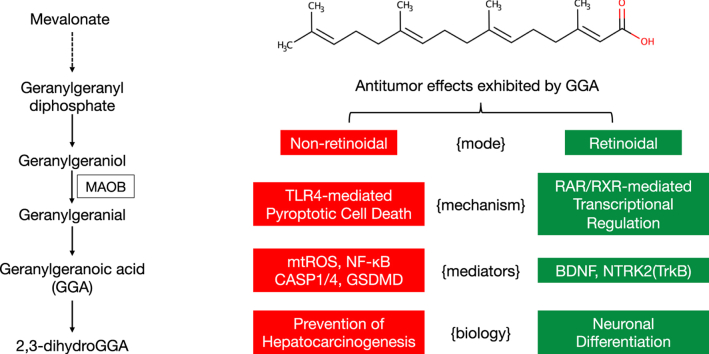

4,5-didehydrogeranylgeranoic acid (4,5-didehydroGGA [XII] or peretinoin) significantly suppressed the incidence of second primary hepatoma up to 5 years after 600 mg daily for 1 year in patients after radical hepatoma surgery in a randomized, placebo-controlled clinical trial (1, 2). Peretinoin, as analogous to its name, is a synthetic chemical developed as one of the retinoids, but we need to go back in history to properly understand its mother compound, geranylgeranoic acid (GGA) [XI].

## Hildebrandt Acid

**Fig. 1:**
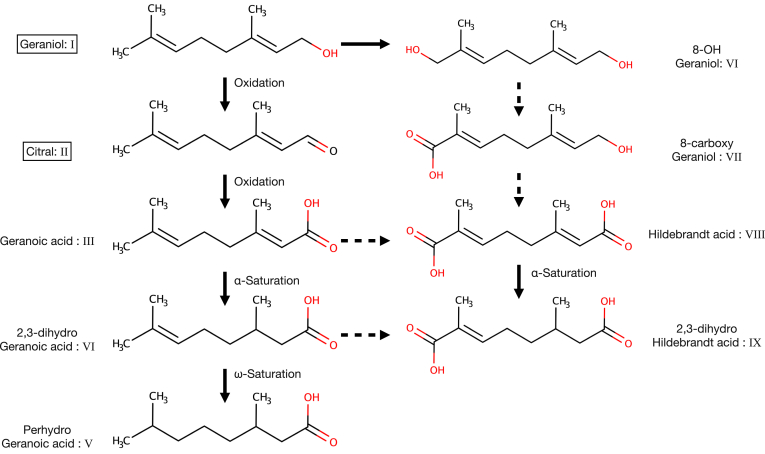
Urinary monoterpenoid metabolites found in mammals to which citral or geraniol had been given orally. The figure shows the metabolites detected in urine and their metabolic relationships after oral administration of geraniol and citral (circled by squares) as exogenous chemicals to mammals such as rabbits and rats. Citral is a collective term that covers two geometric isomers; the E-isomer geranial (*trans*-citral) and the Z-isomer neral (*cis*-citral). The chemical structure of *trans*-citral is shown here. Each enzymatic reaction by a single enzyme is indicated by a solid arrow, and each enzymatic reaction involving multiple enzymes is indicated by a dashed arrow.

In 1900, Hildebrandt, who was studying the pharmacological effects of terpenes, reported the appearance of dicarboxylic acid derivatives of terpenes in the urine of rabbits treated with citral [II] (3). More than 30 years later, Kühn discovered that the substance reported by Hildebrandt was a dicarboxylic acid with a carboxy group at the ω-end of geranoic acid [III] (GA; also written “geranic acid”, but here we use GA as recommended by Kühn) and named the compound "Hildebrandt acid" (4). Hildebrandt acid [VIII] is a compound identified as a metabolite of citral [II], a foreign substance in the body. The discovery of Hildebrandt acid [VIII] in rabbit urine indicates the presence of an enzyme system in animal cells that oxidizes acyclic monoterpenoid alcohols to carboxylic acids. It has recently been shown that geraniol [I], which is often added to perfumes, deodorants, and cosmetics as a rose fragrance ingredient, can be administered to detect GA [III] and Hildebrandt acid [VIII] in human urine and screen for environmental substances using LC-MS/MS technique (5). Figure 1 shows acyclic monoterpenoids previously detected in mammalian urine and their possible metabolic relationships (6).

Hildebrandt acid [VIII] and GA [III] are excreted in urine as compounds that monitor bioexposure to geraniol [I] and citral [II] as environmental substances, but considering that geraniol [I] is an endogenous metabolite, these two organic acids may be physiological metabolites (7). Popjak's group has shown that dimethylallyl diphosphate (DMAPP: C_5_), geranyl diphosphate (C_10_), and farnesyl diphosphate (FPP: C_15_), diphosphate intermediates from mevalonic acid (MVA) to squalene, which are important intermediates in cholesterol synthesis, are all enzymatically dephosphorylated to dimethylallyl alcohol, geraniol [I], and farnesol, respectively. Furthermore, these isoprenols are oxidized by alcohol and aldehyde dehydrogenases to dimethylacrylate, GA [III], and farnesoic acid (FA) in cell-free experiments and mice (8, 9). The results shown in the in vivo experimental system are particularly noteworthy. In particular, of the radioactivity taken up by the liver from the administered radioactive MVA, 11% was in the steroid fraction, 46% in squalene, 4% in allyldiphosphate, 16% in free prenols, and 23% in prenoic acids. At the time, the question of interest was whether the oxidized metabolites of these intermediates of squalene synthesis were intermediates of squalene synthesis or of a separate pathway. As is clear from subsequent developments, there was a report (9) that high concentrations of FA inhibited cholesterol synthesis by inhibiting mevalonate kinase, but not much attention was paid to other isoprenoic acids.

Some of the acyclic monoterpenoid acids described above are precisely the excretions found in human urine and are metabolites of foreign substances such as additives found in cosmetics and foods, some of which may be catabolic degradation products of internal metabolites. So, can we say that all of these acyclic isoprenoid oxidation products are degradation products? Or are they all simply excretions?

## Discovery of GGA in Animals

GGA [XI] is one of the acyclic diterpenoid acids, which we reported to be present in several medicinal herbs (10). At the time, we thought we were the first to report natural GGA [XI], but reports of GGA [XI] as a metabolite in living organisms can be traced back another quarter century (11).

### Geranylgeranyl diphosphate

**Fig. 2:**
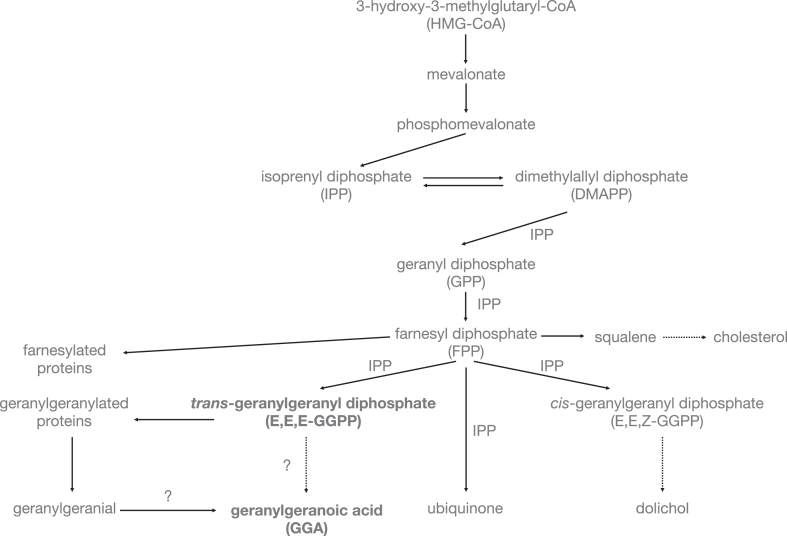
Biosynthesis of GGPP in animal cells and GGPP-derived metabolites. The figure shows the metabolic pathways of the major isoprenoids biosynthesized from HMG-CoA in animal cells, such as cholesterol, dolichol, and ubiquinone, as well as the possible metabolic pathways of geranylgeranyl diphosphate (GGPP).

In mammals, except for the isoprenylation of proteins, two well-known metabolic pathways involving FPP are the biosynthesis of steroids (steroid pathway) and the biosynthesis of isoprenoids, a linear chain of 4–21 isoprene units (nonsteroidal pathway) (Fig. 2). The former is a squalene-mediated pathway in which two FPPs are condensed tail-to-tail, and the latter is a biosynthetic pathway for acyclic isoprenoids of various lengths in which isoprenyl diphosphate (IPP: C_5_) is sequentially condensed onto FPP. The nonsteroidal pathway can be further divided into two pathways: the pathway in which dolichols are synthesized by linking isoprene units in the *cis* configuration and the pathway in which ubiquinone side chains are synthesized by linking isoprene units in the *trans* configuration. The former dolichol is formed by *cis*-prenyltransferase to form a polyprenol with 15–17 isoprene units of IPP linked to FPP in a *cis* configuration, and the polyprenol undergoes α-oxidation by steroid 5 alpha-reductase 3 (SRD5A3) and converted to dolichols (12). The latter CoQ_10_ isoprenyl side chain is synthesized by decaprenyl diphosphate synthase (PDSS1/PDSS2 heterotetramer enzyme) as an isoprenoid with 10 isoprene units using IPP and DMAPP as substrates (13). GGPP, which has only one additional isoprene unit linked in *trans* configuration to FPP, can exist as an intermediate in the biosynthesis of the isoprenyl side chain of CoQ_10_, but GGPP is known to be specifically biosynthesized independently of CoQ_10_ biosynthesis.

In 1981, Sagami *et al.* discovered and purified GGPP synthase from pig liver (14, 15). GGPP synthase uses FPP and IPP as substrates to produce *E,E,E*-GGPP (all-t*rans* GGPP: C_20_), but the biological significance of this GGPP was unknown until geranylgeranylated proteins were discovered (16, 17). GGPP, along with FPP, is now well known as an isoprenoid donor for protein isoprenylation (18). However, as noted by Dallner's group, GGPP synthase activity in rat brains is almost 100 times greater than protein geranylgeranyltransferase activity (19). GGPP may have a different metabolic pathway than protein-geranylgeranylation or may be required for other cellular processes that have not yet been elucidated.

### C_20_-prenoic acid

Interestingly, several compounds that appear to be metabolites of GGPP were also reported around the time of the discovery of GGPP synthase. In 1983, radiolabeled MVA ([2-^3^H]-MVA) was incubated with bovine retinal homogenate and then analyzed for radioactivity (11). The major part of the labeling was incorporated into the saponifiable fraction (20%–40%) rather than into the steroidal fraction (3%–22%), and the majority (90%) of the saponifiable fraction was not C_15_-prenoic acid FA but C_20_-prenoic acid GGA and component IV (the authors at the time considered the possibility of a *cis*-isomer of GGA, but component IV could also be 2,3-dihydroGGA [XIII]). They made similar observations with a tissue culture system of the bovine retina (20). Assuming that C_20_-prenoic acid is an abortive metabolite from an intermediate in the biosynthesis of the CoQ_10_ side chain, it is curious that the formation of C_25_-, C_30_-, C_35_-, C_40_-, and C_50_-prenoic acid has not been reported. Radiolabeled 2,3-dihydroGGA [XIII] and GGA [XI] were also detected in the saponified fraction of triglycerides when [2-^14^C]MVA was incubated in an in vivo culture system of the invertebrate *Schistosoma mansoni* (21). Whole genome analysis of this parasite reported that it lacks the gene for squalene synthase, an essential enzyme in the steroid synthesis system (22). Other than these few early reports, the formation of C_20_-prenoic acids in animal tissues has received relatively little attention.

## Cancer-Preventive Effects of GGA as an Acyclic Retinoid

### Acyclic retinoids

**Fig. 3:**
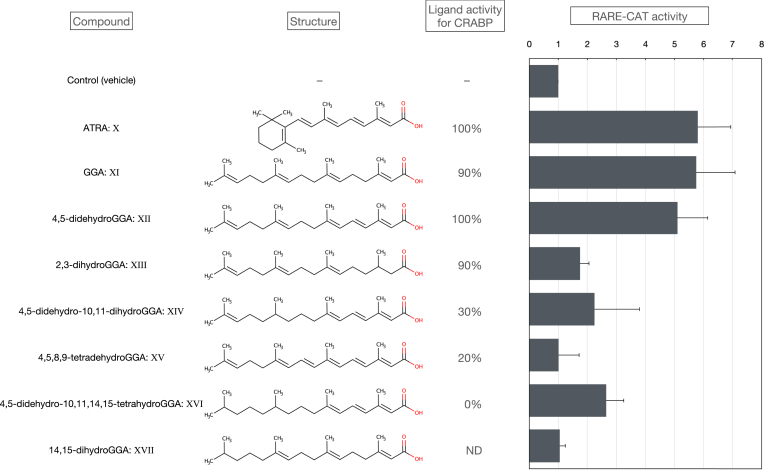
Reporter assay of synthetic GGA derivatives using RARE/CAT (slightly modified from Ref. (26)). The ligand activity to the cellular retinoic acid binding protein (CRABP) was based on the radioactivity of ^3^H-all-*trans* retinoic acid (^3^H-ATRA) bound to CRABP and replaced by a 10-fold molar excess of ATRA. That is, the radioactivity substituted by each synthetic GGA derivative in 10-fold molar excess is shown relative to 100 of ATRA. In addition, plasmid (RARE-CAT), a recombinant prokaryotic chloramphenicol acetyltransferase (*CAT*) gene downstream of retinoic acid response element-β (RARE), was introduced into the human hepatoma-derived cell line HuH-7. After treatment with various retinoids (1 μM each), CAT enzyme activity was measured and expressed as RARE-CAT activity. The RARE-CAT activity of cells treated with each compound is shown relative to the RARE-CAT activity of cells treated with ethanol (vehicle alone). GGA, geranylgeranoic acid.

Since Saffiotti *et al.* reported that the induction of tracheobronchial squamous metaplasia by intratracheal injection of the carcinogen benzo[a]pyrene was inhibited by intragastric administration of vitamin A (retinyl palmitate) (23), cancer chemoprevention and differentiation induction therapy with retinoids became widely advocated (24, 25). Retinoids exert their effects by regulating gene expression by acting as ligands for the well-known nuclear transcription factors, retinoid receptors (retinoic acid receptor [RAR] and retinoic acid receptor [RXR]). Using a plasmid assay with a reporter gene inserted downstream of the retinoid response sequences, GGA [XI] and its 4,5-didehydro derivative [XII] were found to exhibit ligand activity similar to natural retinoids such as all-*trans* retinoic acid (ATRA) [X], as shown in Fig. 3, and 9-*cis* retinoic acid (RA) (26). Thus, compounds that do not have a cyclic structure but can be ligands for retinoid receptors or transcriptional regulators of target genes were named "acyclic retinoids" by Muto and Moriwaki (27). Thus, our research on the antitumor effects of retinoids led us to develop a special interest in acyclic retinoids, which do not exhibit the side effects that retinoids have (1, 2).

Phytanic acid is a well-known oxidative metabolite of phytol, a microbial metabolite of chlorophyll, and has also been reported to be a ligand for RXR. Thus, phytanic acid is also a member of the acyclic retinoid family. However, there are few reports of in vivo studies on the cancer-preventive effects of phytanic acid (28).

### Antitumor actions of acyclic retinoids

Peretinoin (4,5-didehydroGGA [XII]), an acyclic retinoid, was observed to inhibit cell proliferation, upregulate albumin (*ALB*) gene expression, downregulate α-fetoprotein (*AFP*) gene expression, and induce differentiation into hepatocytes when added to a culture system of human hepatoma-derived cell lines (29). Indeed, 9-*cis* RA showed the same effect as 4,5-didehydroGGA [XII], and ATRA [X] similarly decreased *AFP* gene expression, but unlike 4,5-didehydroGGA [XII] and 9-*cis* RA, ATRA [X] also decreased *ALB* gene expression. Thus, it was suggested that the effect of inducing differentiation into hepatocytes is not necessarily common to retinoids.

An acyclic retinoid of 4,5-didehydroGGA [XII] has been reported to induce the differentiation of tumor cells such as leukemia cells and neuroblastoma as well as hepatoma cells. ATRA [X], a natural retinoid, is known to induce the differentiation of acute promyelocytic leukemia (APL) cells into granulocytes, leading to complete remission when taken by patients with APL (30). When primary cultured leukemia cells from APL patients were treated with the acyclic retinoid, a concentration-dependent induction of differentiation was observed, similar to ATRA [X] (31). The acyclic retinoid seems to act exactly as a retinoid.

The differentiation-inducing effects of ATRA [X] on human neuroblastoma-derived cell lines date back to 1981 (32). When neuroblastoma cells are treated with ATRA [X], it is observed that their proliferation is inhibited, their invasiveness is attenuated, and morphological neuron-like projections are formed irreversibly. Furthermore, ATRA [X] treatment restores brain-derived neurotrophic factor (BDNF) dependence of neuroblastoma cells, and a BDNF-dependent increase in amyloid precursor protein (*APP*) gene expression was also observed (33). 4,5-didehydroGGA [XII] also inhibited the proliferation of neuroblastoma cells similarly to ATRA [X], and morphologically, the formation of protrusions more than twice the cell body diameter was observed (34). Subsequently, we observed that another acyclic retinoid, GGA [XI], like ATRA [X], also inhibited the proliferation of the neuroblastoma-derived cell line SH-SY5Y cells, induced neuron-like morphological changes, and significantly induced the expression of the neurotrophic receptor tyrosine kinase-2 (*NTRK2* or *TrkB*) gene, a putative BDNF receptor (35). The cells expressed retinoid receptor proteins of RARα,β,γ and RXRα,γ, and ATRA [X] or GGA [XI] treatment markedly upregulated RARβ expression and downregulated RXRα expression. Thus, we suggest that these two retinoid receptor subtypes expressed in SH-SY5Y cells are implicated in the biological effects of ATRA [X] and GGA [XI]. Acyclic retinoids such as GGA [XI] and 4,5-didehydroGGA [XII] could regulate the expression of differentiation-specific genes in their respective cells via transcription factors such as RAR and RXR as well as ATRA [X] and 9-*cis* RA, leading tumor cells to differentiated cells.

## Natural GGA Found in Plants

We reasoned that if GGA [XI] is a true acyclic retinoid, it could be biosynthesized in plants. As a start, we examined whether GGA [XI] could be detected in lipid extracts of medicinal herbs used in traditional medicine, such as Ayurveda, to cure liver diseases (10). As a result, we were able to identify and quantify GGA [XI] contents from turmeric, Schisandra, licorice, and Indian gooseberry by LC-MS. We then explored several commercial food products and detected GGA [XI] in curry powders, dried parsley flakes, fresh broccoli, and azuki beans (unpublished; YS), but not in polished and brown rice (36).

Since it was shown that turmeric is relatively rich in GGA [XI] (10), we analyzed how the concentration of GGA [XI] in the blood varied after ingestion of commercial turmeric tablets (37). The LC/MS peak of GGA [XI] was detected in the plasma of seven healthy subjects (4 males and 3 females, aged 20–25 years) before ingestion of turmeric tablets, and its concentration ranged from 7.5 to 15.5 ng/ml. Two hours after ingestion of the turmeric tablets, the blood GGA concentration increased by about 1.5 times the basal level and remained at that level until 4 h later but returned to the original value after 8 h. In other words, GGA [XI] is detected physiologically in our human blood, and when GGA [XI] is ingested as a component of food, it is absorbed in the intestine and appears in the blood.

If natural GGA [XI] were found only in plants and this compound were found to be essential for life, the definition of a true acyclic retinoid vitamin could be applied to GGA [XI]. However, since GGA [X] is synthesized also in animal cells (11), this definition is not valid. We next decided to explore the biosynthesis of GGA [XI] in mammals in depth.

## Re-Discovery of Mammalian GGA

### Endogenous GGA

From 1995 to the present, we have been studying the antitumor effects of GGA [XI], especially the mechanism of cell death induction, using a culture system of human hepatoma-derived cell lines. Therefore, we collected HuH-7 cells, a human hepatoma-derived cell line, before adding GGA [XI] to the culture medium, analyzed their lipid extract by LC-MS, and detected a peak consistent with GGA [XI] (38). The possibility of contamination with GGA [XI] contained in FBS, derived from herbivorous ruminants, in the medium, was considered, but no GGA [XI] was found in lipid extracts of FBS. In addition, the intracellular GGA content varied depending on the state of cell proliferation and density whether subconfluent, confluent, or over-confluent. Therefore, we analyzed several cell lines in a subconfluent state and found that GGA content (ng/g wet wt) was about 3.9 in HuH-7 cells, 5.2 in PLC/PRF-5 cells, 3.2 in HepG-2 cells, and 17.7 in HeLa cells, a human cervix cancer-derived cell line. And GGA [XI] was below the detection limit (0.5 ng/g wet wt) in human neuroblastoma-derived cell lines IMR-32 and SH-SY5Y cells (38).

Trace amounts of GGA [XI] were detected by the single mass (m/z[-] = 303.4) signal of molecular ions (deprotonated ions [M-H]^-^ to be precise), making difficult a baseline separation from arachidonic acid, a structural isomer of GGA [XI] that is present in large amounts in animal samples. Therefore, the GGA isomers were then separated using both molecular and fragment ions. Indeed, we subsequently switched to a tandem mass signal detection method using a combination of molecular ion (m/z[-] = 303.4) and fragment ion (m/z[-] = 98.1) and quantified GGA [XI] ([m/z[-] 303 —> 98) separately from arachidonic acid ([m/z[-] 303 —> 259) (39).

Then, we analyzed endogenous free GGA [XI] in each organ of experimental animals (male Wistar rats) by this tandem mass (LC-MS/MS) method. As a result, endogenous free GGA signals were detected in all organs analyzed, especially in the liver at the highest concentration. Apart from the liver, relatively high concentrations of GGA [XI] were detected in the genital organs (testes, prostate, and seminal vesicle) and brain (cerebrum and cerebellum) (39). These are in agreement with the increased reproductive index (RI) in in vivo animal studies (SAM and C3H/HeN mice) in which GGA [XI] was administered orally (40, 41) and with the increased expression of BDNF in the hippocampal dentate gyrus and Cornu Ammonis regions of 1-week-old mice born to mothers which were given GGA [XI] orally (40).

## Biosynthesis of GGA in Human Hepatoma Cells

In mammals, the liver is an active organ in MVA metabolism. Therefore, we analyzed the metabolism of MVA to GGA [XI], which Fliesler & Schroepfer (11, 20) reported in retinal cells as described above, using a culture system of the human hepatoma-derived cell line HuH-7. It was shown that the administration of pravastatin, an inhibitor of HMG-CoA reductase, depleted endogenous GGA in HuH-7 cells within 2 days, and GGA content was restored by mevalonolactone (MVL) supplementation (39). We also found that blocking the main channel of MVA metabolism by adding zaragozic acid A (ZAA or squalestatin), an inhibitor of squalene synthase, resulted in a 10- to 15-fold increase in endogenous GGA [XI] over 3 days. Since the substrate of squalene synthase is FPP, it is possible that the accumulation of FPP by ZAA treatment also increases the flow to GGPP and the accumulation of GGPP enhances the production of GGA [XI].

### Isotopomer spectral analysis (42)

After blocking the synthesis of squalene, a major channel of MVA metabolism, with ZAA treatment and further inhibiting the synthesis of endogenous MVA with pravastatin, stable isotope ^13^C-labeled MVL ([1,2-^13^C_2_]MVL that will be intracellularly metabolized to 5-^13^C-IPP or 5-^13^C-DMAPP) was added and HuH-7 cells were cultured for 48 h; ^13^C_4_-labeled GGA [XI] was detected as the major GGA [XI]. Then, after the addition of ^13^C-MVL, intracellular GGA [XI] was chronologically detected by LC-MS/MS, and the combination of the mass number of the molecular ion (m/z = 303–307 corresponding to ^13^C_0_-GGA–^13^C_4_-GGA) and that of the fragment ion (m/z = 98 or 99 corresponding to no or one ^13^C in the α-isoprene unit of GGA) was analyzed. The eight isotopic isomers (isotopologs and isotopomers) of GGA [XI] could be differentially tracked by this method (39). Then, by setting MVA as a monomer that is incorporated into its tetramer, GGA [XI] (actually IPP or DMAPP corresponds to the monomer) and assuming that the intracellular MVA concentration is constant throughout the experiment, isotopomer spectral analysis (ISA) can be applied even in nonequilibrium conditions.

ISA of intracellular GGA [XI] was performed using HuH-7 cells and ^13^C-MVL. Under the condition that intracellular MVA was kept at the physiological concentration (2.5 mM) and dilution rate *D* with ^13^C-MVL was kept constant at 0.766–0.768 during the experimental period, *g*(t), the ratio of ^13^C-GGA [XI] to the endogenous GGA [XI] after t h of ^13^C-MVL administration was 0.56 at 6 h, 0.88 at 12 h, and 0.9 at 24 h and remained constant thereafter until 72 h. During this period, there was no change in endogenous GGA concentration, and nearly 90% of endogenous GGA [XI] was replaced by newly biosynthesized GGA [XI] at 12 h (39). In other words, GGA [XI] appears to be an intermediate metabolite that is biosynthesized from MVA but metabolized relatively quickly to other compounds. Hepatic GGA [XI] is an endogenous lipid with a rapid metabolic turnover. Thus, GGA [XI] is an acyclic diterpenoid acid, but it does not appear to be an end product of degradation, at least not like the acyclic monoterpenoid acids (see Fig. 1) excreted in human urine described at the beginning of this review.

**Fig. 4:**
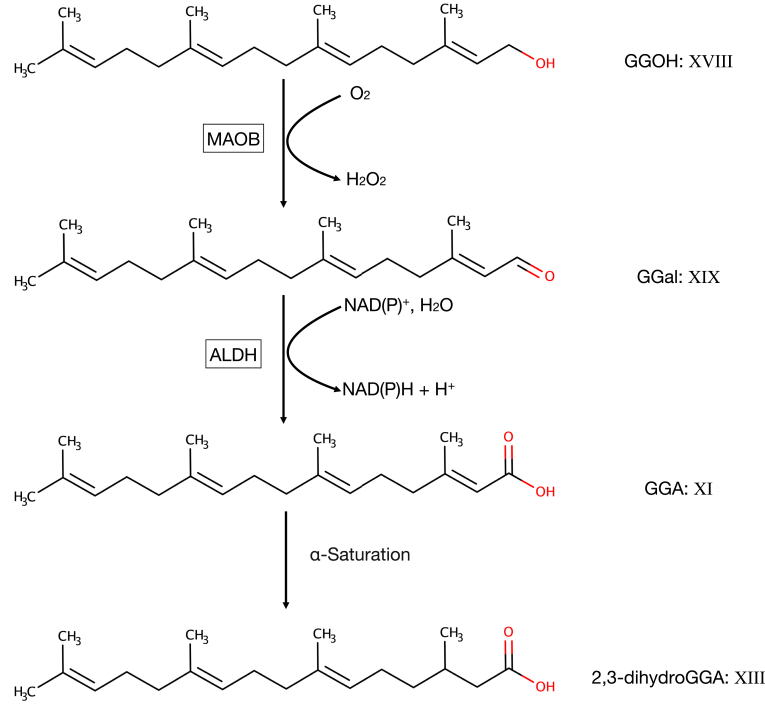
Hepatic MAOB is involved in the oxidation of GGOH to GGal along with CYP3A4, the backup enzyme for MAOB during GGA synthesis. The enzymatic reaction from GGOH to geranylgeranyl aldehyde (GGal) is NAD(P)^+^-independent in human hepatoma cells and rat liver homogenates and involves the consumption of molecular oxygen (38), and MAOB inhibitor treatment reduces intracellular GGA content. In addition, it has been shown that hepatic MAOB is involved in the oxidation reaction of GGOH to GGal, as MAOB inhibitor treatment decreased the intracellular GGA content and knockdown of the *MAOB* gene using a specific siRNA-inhibited GGA synthesis from GGOH (43). On the other hand, intracellular GGA content was maintained at the same level in MAOB-KO cells as in wild-type cells, indicating that CYP3A4, as a backup enzyme of MAOB, is involved in the oxidation of GGOH to GGal, as shown by experiments using inhibitors and siRNA (44). The oxidation of GGal to GGA is NAD(P)^+^ -dependent and is catalyzed by ALDH, a nonspecific enzyme. Isotopomer spectral analysis showed that GGA is further metabolized to 2,3-dihydroGGA (39), and endogenous 2,3-dihydroGGA was consistently detected in higher concentrations than GGA in each organ of Wistar rats (39). GGA, geranylgeranoic acid; MAOB, monoamine oxidase B; ALDH, aldehyde dehydrogenase.

As described below, more 2,3-dihydroGGA [XIII] than GGA [XI] was detected in each organ of rats. Therefore, we performed ISA of 2,3-dihydroGGA [XIII] with the same samples used for ISA of GGA [XI]. As a result, the *g*(t) value of 2,3-dihydroGGA [XIII] at 12 h after ^13^C-MVL administration was 0.68, which was smaller than that of GGA [XI]. And the *g*(t) value of 2,3-dihydroGGA [XIII] caught up with that of GGA [XI] after 72 h, suggesting metabolism (α-saturation) from GGA [XI] to 2,3-dihydroGGA [XIII] (Fig. 4) (39), which has already been reported to be synthesized from MVA in the parasite (21), as mentioned above.

## Oxidation of Geranylgeraniol Requires Molecular Oxygen

### From GGPP to GGOH

The next question was what enzymatic reactions are involved in the metabolism from GGPP to GGA [XI]. Since a specific GGPP pyrophosphatase had already been reported that specifically uses GGPP but not FPP as a substrate in rat hepatocytes (45), it was suggested that the enzyme converted GGPP to geranylgeraniol (GGOH) [XVIII] in a one-step reaction. However, a recent report that type 1 polyisoprenoid diphosphate phosphatase (PDP1 or phospholipid phosphatase 6) is involved in the regulation of intracellular GGOH concentrations suggested that the dephosphorylation of GGPP to GGOH [XVIII] is catalyzed by the phosphatase rather than pyrophosphatase, indicating that phosphate groups may be sequentially released from GGPP (46). However, PDP1 was originally identified as an enzyme that converts presqualene diphosphate to presqualene monophosphate (47) and also acts equally on geranyl diphosphate, FPP, and GGPP to produce geraniol [I], farnesol, and GGOH [XVIII], respectively (48). Whether the enzyme involved in the dephosphorylation of GGPP is the former pyrophosphatase or the latter phosphatase, the existence of the enzymatic reaction(s) from GGPP to GGOH [XVIII] in mammalian cells is confirmed.

### From GGOH to GGA

Therefore, we focused our analysis on the oxidation reaction from GGOH [XVIII] to GGA [XI]. Initially, we assumed that the reactions would be catalyzed by enzymes that exhibit relatively broad specificity, such as alcohol dehydrogenase (ADH) and aldehyde dehydrogenase, which are known to catalyze the metabolism from retinol to retinoic acid via retinal. However, the metabolism from GGOH [XVIII] to geranylgeranyl aldehyde (GGal) [XIX] did not require the addition of NAD^+^ in a rat liver homogenate system, and the oxidation from GGal [XIX] to GGA [XI] was found to be NAD^+^-dependent (36). Moreover, consumption of molecular oxygen was observed in the oxidation reaction from GGOH [XVIII] to GGal [XIX], indicating that the reaction from GGOH [XVIII] to GGal [XIX] is not a dehydrogenation reaction but a reaction catalyzed by an oxidase that requires oxygen as a substrate and that the enzyme activity is localized in the mitochondrial fraction (38). It was also shown that tranylcypromine (TCP), an inhibitor of monoamine oxidase A/B (MAOA/B), suppresses the synthesis of GGA from GGOH [XVIII] and that human recombinant MAOB, but not MAOA, protein efficiently produces GGal [XIX] from GGOH [XVIII] (38). Subsequently, we demonstrated that siRNA-mediated knockdown of the *MAOB* gene reduced endogenous GGA content in HuH-7 and Hep3B, indicating that the oxidation of GGOH [XVIII] to GGal [XIX] is catalyzed by MAOB in human hepatocytes (Fig. 4) (43).

### GGA in *MAOB*-KO cells

However, contrary to our expectations, when *MAOB*-KO cells were established in Hep3B cells by the CRISPR/Cas-9 system, the endogenous GGA concentration in the KO cells did not differ from that in WT Hep3B cells (43). Moreover, since the endogenous GGA concentration did not change after TCP treatment of the KO cells, it was unlikely that any residual MAOB was involved in the maintenance of endogenous GGA levels, suggesting that an enzyme other than MAOB may be induced as a backup for GGA synthesis. Interestingly, when the human *MAOB* gene was introduced into these KO cells, the endogenous GGA concentration did not change, but the endogenous GGA concentration decreased upon TCP or *MAOB* siRNA treatment. In other words, even in *MAOB*-KO cells in which some backup system was induced, MAOB was shown to be preferentially used for GGA synthesis when the *MAOB* gene was reintroduced (43).

So what could replace the MAOB enzyme in the backup system for GGA synthesis in *MAOB*-KO cells? We searched for that possible enzyme. Our previous studies showed that GGOH oxidase activity is present in the microsomal fraction as well as in the mitochondrial fraction where MAOB is localized, but not detected in the cytosol fraction, in rat liver (36) and HuH-7 cells (37). Endo *et al.* (49) reported that ADH1A, which is localized in the cytosol fraction, generates GGal [XIX] using GGOH [XVIII] as a substrate, but even when *ADH1A* gene expression was knocked down using siRNA specific for *ADH1A*, the endogenous GGA levels in Hep3B/*MAOB*-KO cells did not decrease (43). This is consistent with our previous finding that no enzymatic activity to oxidize GGOH [XVIII] to GGal [XIX] was detected in the cytosolic fraction (37). On the other hand, a group of cytochrome P450 enzymes localized in the liver microsomal fraction are known to oxidize isoprenols such as geraniol [I] (50) and retinol (51). Therefore, we decided to search for members of the cytochrome p450 enzyme family that are induced in *MAOB*-KO cells and oxidize GGOH [XVIII].

### CYP3A4, a backup enzyme for MAOB

Expectedly, treatment of *MAOB*-KO (Hep3B/*MAOB*-KO) cells with the cytochrome P450 enzyme activity pan-inhibitors 1-aminobenzotriazole (ABT) or bergamottin (BG) resulted in a concentration-dependent decrease in endogenous GGA levels (IC_50_ 10.9 μM for ABT, 7.5 μM for BG) (44). On the other hand, in the WT (Hep3B/*MAOB*-WT) cells expressing the *MAOB* gene, no decrease in endogenous GGA concentration was observed with these inhibitors, indicating that cytochrome P450 enzymes are involved in the synthesis of endogenous GGA [XI] only in *MAOB*-KO cells. Therefore, we first analyzed whether cytochrome P450 enzymes are upregulated in *MAOB*-KO cells; Hep3B/*MAOB*-WT cells express *CYP1A1*, *CYP1A2*, *CYP2A6*, *CYP2B6*, *CYP3A4*, and *CYP3A5* genes among cytochrome P450 enzymes. In Hep3B/*MAOB*-KO cells, the expression of *CYP3A4* was upregulated by about 3-fold, but the expression of other *CYP*s did not change. Knockdown of *CYP3A4* gene expression in *MAOB*-KO cells using specific siRNA significantly decreased endogenous GGA levels. Furthermore, when recombinant human CYP3A4 protein was mixed with GGOH [XVIII], GGal [XIX] was produced in a GGOH concentration- and reaction time-dependent manner. The inhibitory activity of ABT and BG against the recombinant CYP3A4 (IC_50_ 10 μM and 4.5 μM, respectively) is in good agreement with the aforementioned inhibitory effect of the two inhibitors on GGA concentration in *MAOB*-KO cells. These results indicate that CYP3A4 acts as a backup enzyme for MAOB in *MAOB*-KO cells and maintains the intracellular concentration of GGA [XI]. Why the intracellular GGA concentration must be maintained even with the backup enzyme is a question that needs to be resolved.

## Prevention of Spontaneous Hepatoma in C3H/HeN Mice by GGA

### Age-dependent decrease in liver GGA content

Male C3H/HeN mice are known to spontaneously develop hepatoma at a high rate after 2 years of normal rearing (52). Furthermore, it has been reported that oral administration of 4,5-didehydroGGA [XII] (50 μg/mouse) to male C3H/HeN mice at 12 months of age greatly reduced the number of cases of hepatoma detected at 23 months of age (53, 54). Therefore, we hypothesized that the liver GGA content of C3H/HeN mice declines with aging and that when the value declines past a putative threshold, it may favor for the development of hepatoma. Indeed, hepatic GGA concentrations in C3H/HeN mice decreased with age, and GGA [XI] could not be detected in the livers of 93-week-old mice (55). Hepatic GGA levels in male C3H/HeN mice showed a two-stage decrease with age. In the first stage, hepatic GGA levels decrease by approximately 30% from 18 to 25 weeks of age, and in the second stage, they decrease rapidly and become undetectable from 60 to 93 weeks of age (55).

### Age-dependent decrease in hepatic *MAOB* gene expression

Since the age-related decrease in hepatic GGA content is thought to be due to a decrease in GGA synthesis, the expression level of the *MAOB* gene was analyzed. Since long ago, *MAOB* gene expression has been shown in studies in human postmortem brains, where MAOB activity generally increases with age and MAOA activity remains largely unchanged (56). In rodents, as in humans, MAOA activity changes very little with age, and MAOB activity is known to increase with age in all brain regions except the brainstem (56). Although most enzyme activities are characterized by a decrease with age, MAOB may be a rare exception. Indeed, *MAOB* mRNA levels increased with aging in the brains of C3H/HeN mice. However, although hepatic *MAOB* mRNA levels increased progressively until about 18 weeks of age, they decreased with age to about one-third of their maximum level at 93 weeks of age (55). A significant positive correlation between liver *MAOB* mRNA levels and hepatic GGA levels was detected, suggesting that the age-related decrease in hepatic GGA levels was due to decreased expression of the liver *MAOB* gene.

### Prevention of spontaneous hepatoma by oral administration of GGA

Assuming that a decrease in hepatic GGA levels in C3H/HeN mice would allow spontaneous hepatocarcinogenesis to develop, we wondered if increasing hepatic GGA levels by administering exogenous GGA [XI] when hepatic GGA levels were decreased would suppress spontaneous hepatocarcinogenesis. We observed that oral administration of GGA [XI] or GGOH [XVIII] or intraperitoneal administration of ZAA to 24-week-old mice significantly increased liver GGA content (55). Therefore, we administered a single oral dose of GGA [XI] to mice at 7 months of age, when liver GGA levels begin to decrease, at 11 months of age, which is 4 months after liver GGA levels stabilize at a low level, or at 17 months of age, when liver GGA levels begin to rapidly decrease further, respectively. Subsequent autopsy of livers at 24 months of age revealed that a single oral dose of GGA [XI] at 11 months of age, as in the case of 4,5-didehydroGGA [XII] described above (53, 54), significantly reduced the detection rate of spontaneous hepatoma (55). A single dose of GGA [XI] at 7 or 17 months of age had no effect on suppressing spontaneous hepatomas, and the detection rate of hepatoma in either group was not significantly different than in the untreated control group.

As mentioned above, GGA metabolism is rapid, so it is unlikely that a single dose of GGA [XI] administered to 11-month-old mice will exert its effects for a long period of time, even up to 24-month-old mice. Therefore, we will discuss the mechanism by which GGA [XI] administered as a single dose at the age of 11 months exerts its effects for more than one year based on experimental observations. Three observations are important in considering the mechanism: 1) liver GGA content in male C3H/HeN mice begins to decrease with age at around 6 months of age, and liver GGA levels remain low during 8–15 months of age and then decrease further until no GGA [XI] becomes detectable at 23 months of age; 2) a single oral administration of GGA [XI] to mice causes a transient increase in liver GGA concentration; and 3) GGA [XI] induces inflammatory cell death in a concentration-dependent manner selectively in tumor cells but not in primary cultured hepatocytes, which is described in detail later. Based on these three things, the possibility that a single dose of GGA [XI] at 11 months of age could cause irreversible changes in C3H/HeN mice is as follows. That is, a single oral administration of GGA [XI] to C3H/HeN mice at the age of 11–12 months when liver GGA levels have decreased with age transiently increases liver GGA levels, which induces inflammatory cell death in all tumor cells (or precancerous cells) developing in the liver at the age of 11 months and eliminates them from the liver so that the spontaneous hepatocarcinogenesis does not occur during the following one year. If GGA [XI] is not administered during this period, existing tumor cells are expected to develop into hepatomas after one year because they will continue to grow, evading cell death due to low liver GGA concentrations in the liver.

On the other hand, GGA [XI] administered as a single dose at the age of 7 or 17 months shows no inhibitory effect on hepatocarcinogenesis. The reason for this, based on the three observations mentioned above, is speculated that GGA [XI] administered at the age of 7 months is ineffective because GGA-sensitive tumor cells have not yet developed, and GGA [XI] administered at the age of 17 months cannot eliminate tumor cells because they have already increased in malignancy and lost GGA sensitivity. However, we believe that a detailed understanding of the mechanism of GGA inhibition of hepatocarcinogenesis will have to wait until the mechanism of spontaneous hepatocarcinogenesis in C3H/HeN mice is clarified.

## GGA-Induced Cell Death in Hepatoma Cells

### Nonretinoidal function

Now the fact that we know that GGA [XI] is synthesized de novo in mammalian hepatocytes, that its synthesis decreases in an age-dependent manner, and that administration of exogenous GGA [XI] inhibits the development of spontaneous hepatoma in vivo, what is the mechanism by which GGA [XI] prevents carcinogenesis? As mentioned above, we were initially interested in the differentiation-inducing effects of retinoids on hepatoma cells and began serious research on the antitumor effects of acyclic retinoids (29). At that time, ATRA [X] was attracting attention as an active metabolite of retinoids, and cellular retinoic acid binding protein (CRABP) was reported as a factor mediating its action (57). GGA [XI] was then found as one of the synthetic retinoids, indexed by its ligand activity against CRABP (58). Later, retinoid receptors (RAR, RXR) were discovered as more reliable mediators of RA activity at the transcription levels (59), and ligand activity against them was considered more essential as an indicator of retinoid biological activity. As already mentioned, GGA [XI] showed ligand activity against CRABP, RAR, and RXR (Fig. 3). GGA [XI] dramatically reduced the number of viable HuH-7 cells in a concentration-dependent manner, but even media containing higher concentrations of GGA [XI] did not alter the number of live primary mouse hepatocytes (60). Notably, this cell death-inducing effect on hepatomas, detailed below, is specific to GGA [XI] and not to retinoids such as ATRA [X] and 9-*cis* RA (61). Therefore, we do not believe that GGA-induced cell death is directly related to retinoid-binding proteins such as CRABP, RAR, and RXR. In other words, the cell death-inducing effects of GGA [XI] that may be directly related to its carcinogenesis inhibitory effects are considered to be nonretinoid effects.

### Apoptotic cell death

**Fig. 5:**
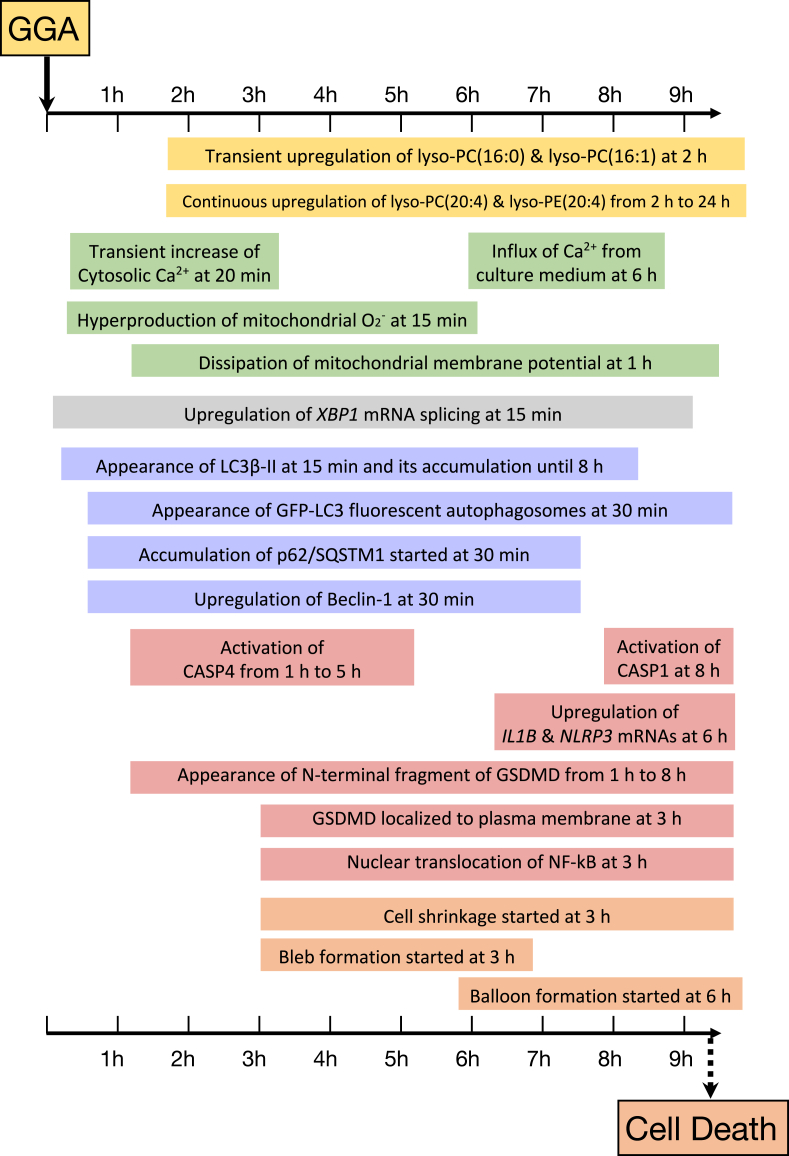
Timeline of the cellular events during GGA-induced cell death of human hepatoma HuH-7 cells. The increase in intracellular lyso-PC and lyso-PE after GGA treatment was detected by metabolomics analysis without preconception (62). The increases in intracellular calcium ions and mitochondrial superoxide after GGA treatment were quantified in time series by observing a culture system of HuH-7 cells with Fluo-4 AM or MitoSox Red incorporated using a laser scanning confocal fluorescence microscope and collecting live cell images in time-lapse (63). Cellular mRNA levels of *XBP1s* (the spliced form of *XBP1* mRNA), *IL1B*, and *NLRP3* genes were quantified by RT-qPCR, while LC3B-II, p62, Beclin-1, active CASP1, active CASP4, and N-terminal fragment of GSDMD (gasdermin D) were measured by Western blotting (63). Laser scanning immunofluorescence confocal microscopy observed subcellular localization of GSDMD and NF-κB (63). GGA, geranylgeranoic acid; lyso-PC, lysophosphatidylcholine; lyso-PE, lysophosphatidylethanolamine.

When GGA [XI] is added to the medium at concentrations of 1–20 μM to several human hepatoma-derived cell lines, including HuH-7, HepG-2, Hep3B, and PLC/PRF-5, cell death is induced in approximately 8–10 h (Fig. 5). Initially, GGA-induced cell death was presumed to be due to apoptosis, since the dissipation of mitochondrial inner membrane potential and condensation of chromatin were observed before the cell death, and the cell death was inhibited or delayed by selective inhibitory peptides for caspase-1 (CASP1) or CASP3, respectively (60, 61, 64). However, the subsequent examination did not reveal cytoplasmic leakage of cytochrome *c* from mitochondria or activation of CASP3 (unpublished; KO and YS), which we considered to be different from typical apoptosis.

### Autophagic cell death

Next, we analyzed autophagy, another mode of programmed cell death. Western blot analysis of markers of autophagosomes, LC3β-II, live fluorescence microscopy of GFP-RFP-LC3β-expressing cells, Western blot analysis of p62/SQSTM1 (sequestosome 1), a cargo of autophagosomes degraded by autolysosomes, and electron microscopy of autophagosomes revealed that GGA [XI] induces an incomplete autophagic response, which is mainly characterized by the abnormal accumulation of early autophagosomes and failure of autolysosome formation (65). At that time, we considered that this "incomplete response" of autophagy itself might cause GGA-induced cell death.

Mutant p53 protein stored in the cytoplasm is thought to be involved in the inhibition of autophagy (66). Thus, the observation that mutant p53 protein is stored in the cytoplasm in HuH-7 (Y220C) and PLC/PRF-5 cells (R249S) and rapidly translocated to the nucleus upon GGA treatment (67) suggests that GGA [XI] at least releases mutant p53-mediated inhibition of autophagy.

### Unfolded protein response

Around 2012, ER stress had been reported as one of the triggers of autophagy along with starvation stress. Therefore, after GGA treatment of human hepatoma-derived cell lines, we analyzed three pathways of ER stress-induced unfolded protein response (UPR^ER^); splicing of *XBP1* mRNA on the ER membrane (IRE1 pathway), *DDIT 3* gene transcription level (PERK pathway), and *PDIA4* gene transcription level (ATF6 pathway). As a result, activation of the IRE1 and PERK but not the ATF6 pathway was observed (68). This is similar to the lipid-induced UPR^ER^ caused by high concentrations (sub-mM) of saturated fatty acids (such as palmitic acid (PA) and stearic acid), whereas the tunicamycin-induced UPR^ER^ involves activation of all three pathways (69). GGA-induced UPR^ER^, like lipid-induced UPR^ER^, was suppressed by the coexistence of oleic acid (OA), a monounsaturated fatty acid. Interestingly, the effect of OA cotreatment inhibited not only UPR^ER^ but also GGA-induced incomplete response of autophagy and even GGA-induced cell death, suggesting that GGA-induced UPR^ER^ is the upstream signal, followed by the incomplete response of autophagy downstream, leading to cell death. Activation of ATF4, which is downstream of the PERK pathway in the UPR^ER^ signal, is known to enhance Beclin-1 expression and then Beclin-1 promotes autophagosome formation (70). We reported a rapid upregulation of Beclin-1 by GGA (65), indicating that GGA-induced UPR^ER^ triggers an autophagic response.

We observed rapid loss of cyclin D1 protein upon GGA treatment in several human hepatoma cell lines (HuH-7, PLC/PRF-5, and HepG2) and reported that the mechanism is not transcriptional regulation of the cyclin D1 (*CCND1*) gene but the rapid arrest of its translation (71). At the time of writing that paper, we were unable to explain the mechanism of the translational repression, but it can now be easily explained by assuming that the GGA-induced UPR^ER^ activates the PERK pathway and phosphorylates eIF2α, which in turn stops translation of the *CCND1* mRNA (72).

The induction of UPR^ER^ by GGA [XI] may explain another interesting phenomenon induced by GGA [XI]. Namely, TP53-induced glycolysis and apoptosis regulator (TIGAR), a repressor of the glycolysis, and synthesis of cytochrome C oxidase 2 (SCO2), an assembly factor of mitochondrial respiratory chain complex IV, were rapidly increased upon GGA treatment of HuH-7 cells, and conversion of energy metabolism from glycolysis to respiration was observed (73). Although the mechanism was unknown at the time, the structure-activity relationship of UPR^ER^ induction with GGA derivatives was consistent with that of SCO2 upregulation activity, leading to the hypothesis that UPR^ER^ induction may increase SCO2. Recently, it was shown that UPR^ER^ induction by glucose deprivation or tunicamycin treatment increases the formation of respiratory chain supercomplexes (74). The authors of this paper propose that the mechanism is that, activation of PERK, one of the three UPR^ER^ branches, induces the assembly of respiratory chain supercomplexes through a signaling flow of increased phosphorylation of eIF2α, upregulation of ATF4, and increased expression of supercomplex assembly factor-1. It is possible that the same mechanism operated in GGA-induced UPR^ER^ to increase SCO2.

### Quick induction of UPR^ER^

GGA [XI] was shown to induce so-called "lipid-induced UPR^ER^," which is well known to be induced by lipids such as PA, but it is unclear how GGA [XI] induces UPR^ER^. Therefore, we followed GGA-induced UPR^ER^ immediately after the addition of GGA [XI] using splicing of *XBP1* mRNA as an indicator. UPR^ER^ was already observed 5 min after the addition of GGA [XI] to the medium (unpublished; CI and YS). Since it is currently experimentally impossible to analyze at an earlier time, we have confirmed that the signal of GGA addition reaches the ER membrane within 5 min. In addition, as mentioned above, simultaneous treatment with OA completely suppresses both GGA-induced UPR^ER^ and cell death but not at all when cells are pretreated with OA (1–24 h before GGA addition), that is, when OA is not present outside the cell during GGA treatment, suggesting that OA is not eliminating GGA signal inside the cell but rather eliminating it at the cell surface (68). In other words, we can hypothesize that the point of action of GGA [XI] in UPR^ER^ induction and cell death induction may be near the cell surface.

We, therefore, focused on reports that cell death induction by lipids such as saturated fatty acids like PA, or lipotoxicity, is dependent on the activation of toll-like receptor-4 (TLR4) localized at the cell surface (75, 76). In other words, lipids such as saturated fatty acids and cholesterol induce the UPR^ER^ and stimulate TLR4, which is known to be a receptor that originally recognizes lipopolysaccharides produced by Gram-negative bacteria.

### Direct activation of TLR4 by PA

A possible mechanism by which PA activates TLR4 has been reported (77), in which PA acts directly on TLR4 activation by acting as a ligand for the TLR4/MD-2 complex. According to their docking simulations, five molecules of PA dock into the hydrophobic pocket of MD-2 and activate and internalize TLR4 by cross-linking TLR4 as a dimer and recruiting it to lipid rafts on the cell membrane. Activated TLR4 transduces signals into the cell, which is thought to activate IRE1 localized to the ER and cause splicing of *XBP1* mRNA on the ER (78, 79). The observation that *TLR4*-KO mice do not develop UPR^ER^ even when reared on a high-fat diet strongly supports this idea (80).

However, there is also a report (81) that when radiolabeled stearic acid was used to analyze the binding of the fatty acid to TLR4/MD-2 fusion protein, no evidence of binding was obtained; if PA is directly involved in the activation of TLR4, it may be necessary to consider that PA is not only a ligand for TLR4/MD-2 but also promotes the translocation of TLR4 to the membrane raft. Moreover, the mechanism by which TLR4 activation leads to the activation of IRE1 localized to the ER is still unclear.

### Indirect activation of TLR4 by PA

PA has also been reported to cause UPR^ER^ without requiring activation of TLR4, although it causes TLR4-mediated inflammatory responses (82). One mechanism is that PA taken up by cells is metabolized to lysophospholipids (lyso-PLs) and diacylglycerols (DAGs), which perturb the ER membrane, resulting in the activation of IRE1 and PERK, sensors of ER stress localized in the perturbed membrane domains. PA-induced UPR^ER^ is inhibited by cotreatment with OA, which is thought to be a mechanism by which OA is metabolized into phospholipids and does not activate IRE1 or PERK by maintaining healthy ER membranes (69).

If PA causes UPR^ER^ without TLR4 activation, how is TLR4 activated after PA addition? UPR^ER^ induced by PA metabolites such as lyso-PLs and DAGs activates IRE1, followed by enhanced ceramide synthesis and secretion of ceramide-containing extracellular vesicles, which in turn activate TLR4 (83). In this regard, the recent identification of sphingosine-1-phosphate lyase (SPL) as a substrate of the IRE1 enzyme (84) points to the possibility that phosphorylation of SPL by IRE1 inhibits its activity, resulting in ceramide increase and consequent activation of TLR4.

In any case, it remains an open question as to how PA activates TLR4, although this may vary depending on cellular context (85).

### TLR4 activation by GGA

We now consider the possibility and mechanism of TLR4 activation by GGA [XI] using the activation of UPR^ER^ and TLR4 by PA as a model. Through the use of specific inhibitors and gene knockdown experiments, we were able to show that the two processes essential for the induction of hepatoma cell death by GGA [XI] are UPR^ER^ induction and TLR4 activation, which are suppressed by cotreatment with OA as in the case of PA (63). There is no experimental evidence that GGA [XI] is incorporated into lyso-PLs or DAGs as in the case of PA. However, of note, we recently detected without any preconceptions by comprehensive metabolomics analysis that immediately after treatment of HuH-7 cells with 10 μM GGA [XI], nine molecular species of lyso-PLs are overwhelmingly increased compared to other cellular metabolites (62). Moreover, lysophosphatidylcholine (lyso-PC) containing one of PA (C16:0), palmitoleic acid (C16:1), or arachidonic acid (C20:4) and lysophosphatidylethanolamine containing C20:4 increased most quickly (see Fig. 5), while lyso-PC containing dihomo-γ-linolenic acid (C20:3) increased later. Interestingly, as with the addition of PA, lyso-PC containing PA showed a rapid and transient increase (peaking at 2 h after GGA addition at the latest) followed by a slow increase, reaching the highest concentration among the increased lyso-PLs (62). Therefore, the same mechanism of lipotoxicity exhibited by PA may be at work in the induction of cell death of hepatoma cells by GGA [XI]. In other words, it can be hypothesized that GGA [XI] induces UPR^ER^, which causes activation of IRE1 and PERK by increasing PA-containing lyso-PC in the ER membrane, thereby increasing intracellular ceramide levels and stimulating TLR4 by ceramide-containing vesicles secreted to the extracellular space.

However, unlike the case of PA, GGA [XI] cannot be directly metabolized to PA-containing lyso-PC, which raises the question of what mechanism allows GGA [XI] to increase PA-containing lyso-PC. To answer this question, based on the characterization of the molecular species of the increased lyso-PLs, we suspect that GGA [XI] may be responsible for the decrease in ectonucleotide pyrophosphatase/phosphodiesterase 2 (ENPP2) or autotaxin activity (62). ENPP2, an enzyme that acts on lyso-PLs to produce lysophosphatidic acid, plays a very important role in hepatoma development (86), including the growth of hepatitis B virus (87) and hepatitis C virus (88), and high-grade hepatomas are reported to have high ENPP2 expression (89). Assuming that GGA [XI] is involved in the downregulation of ENPP2, it is very interesting to consider the preventive effect of GGA [XI] on hepatocarcinogenesis.

Therefore, it is possible to assume that GGA increases PA-containing lyso-PCs via downregulation of ENPP2, the increased lyso-PC (C16:0) activates IRE1 on the ER membrane, the activated IRE1 suppresses SPL activity via phosphorylation, resulting in increased ceramide and consequent activation of TLR4. However, even if such an assumption can be made, in experiments with specific inhibitors of TLR4 or gene knockdown, the most upstream signal for GGA-induced cell death in hepatoma is TLR4 activation. Thus, the downregulation of ENPP2 may occur downstream of TLR4 activation signaling by GGA. We have so far not examined the possibility that GGA directly activates TLR4 nor whether GGA-activated TLR4 signaling is involved in the downregulation of ENPP2. In any case, there is no doubt that TLR4 plays an essential role in GGA signaling, as treatment with VIPER, a specific inhibitory peptide of TLR4, or knockdown of the *TLR4* gene with siRNA completely suppresses GGA-induced cell death and UPR^ER^ induction as well as incomplete autophagy responses (63).

### Pyroptotic cell death induced by GGA (Fig. 5)

Although the signal of GGA addition was shown to flow from TLR4 activation to autophagy through lipid-induced UPR^ER^, the mechanism of GGA-induced cell death remained unknown. Therefore, we observed the activation of CASPs, which is directly related to programmed cell death and detected the activation of CASP4 from 1 h to 5 h after the addition of GGA [XI]; along with the activation of CASP4, gasdermin D (GSDMD)-N, the N-terminal fragment of GSDMD was produced, and 3 h after the addition of GGA [XI], immunofluorescence microscopy also confirmed the localization of GSDMD at the plasma membrane (see Fig. 5). Subsequently, rapid activation of CASP1 was observed 8 h after the addition of GGA [XI] (63). In HuH-7 cells, treatment with thapsigargin (an established ER stress inducer through inhibition of the sarco/endoplasmic reticulum Ca^2+^ ATPase) alone, which decreases Ca^2+^ concentration in the ER, activated CASP4 in the same manner as GGA treatment (63). CASP4 is considered to be the enzyme responsible for ER stress-induced cell death (90). Recently, it was shown that the mechanism of CASP4 activation is that mitochondrial-localized calpain 5 (CAPN5) is activated by ER stress and the active proteolytic enzyme CAPN5 cleaves pro-CASP4 (91).

On the other hand, the activation of CASP1 is generally considered to be carried out by the formation of inflammasomes independently of the activation of CASP4. However, during GGA treatment, the activation of CASP1 is also inhibited by the cotreatment with CASP4 inhibitor, suggesting that pyroptosis through a noncanonical pathway in which CASP4 activation is involved in the formation of inflammasomes occurs. Moreover, as shown in Fig. 5, after GGA treatment, increased production of superoxide in mitochondria, nuclear translocation of NF-κB, and increased expression of *NLRP3* and *IL-1B* genes (so-called “priming of inflammasome”) are also induced to follow the activation of CASP4, indicating that the pyroptosis by the canonical pathway is also thought to be progressing.

In considering the mechanism of GGA-induced cell death, it makes sense to consider the following observations: GGA [XI] induced cell death in the guinea pig fibroblast cell line 104C1, but the cell line 104 C/O4C, which is stably expressing the human phospholipid hydroperoxide glutathione peroxidase (*PHGPx* or *GPX4*) gene, became GGA-resistant. Hyperproduction of superoxide in mitochondria was observed in both cell lines upon GGA treatment, but GGA-induced accumulation of peroxides and loss of mitochondrial inner membrane potential were observed only in the 104C cells and not in the 104 C/O4C cells (92). The fact that PHGPx suppresses GGA-induced cell death indicates that there may be a common process with ferroptosis in GGA-induced pyroptotic cell death.

In summary, it was shown that the administration of exogenous GGA [XI] to hepatoma cells acts in an inhibitory manner against hepatocarcinogenesis by inducing pyroptosis, a form of inflammatory cell death, via TLR4, which is upregulated in hepatoma cells (93, 94). It was also shown that increasing the intracellular content of endogenous GGA [XI], one of the endogenous MVA metabolites, with drugs such as ZAA leads to similar cell death in hepatoma cells (39). Future research on GGA targeting is needed for prophylactic strategies against liver cancer.

## Other Biological Functions of GGA

We have described above the inhibitory effects of GGA [XI], one of the MVA metabolites, on hepatocarcinogenesis. However, in addition to this, GGA [XI] also has the potential to act in the reproductive and cranial nervous systems. We had been breeding and rearing senescence-accelerated mice, SAM-P8, characterized by accelerated aging of the brain nervous system and found by chance that rearing with GGA [XI] added to a commercial solid feed for breeding and lactation period significantly increased the number of weaned pups per mating (reproduction index: RI) (40). Subsequently, a similar experiment was conducted in another strain C3H mice, and an increase in RI was observed upon GGA supplementation (41). When endogenous GGA [XI] was quantified in various organs of 5-week-old male Wistar rats, the second highest amount of GGA [XI] was found in the testes and epididymis (39). This suggests that the increase in RI associated with GGA supplementation may be due to GGA-induced enhancement of sperm maturation and fertility. Indeed, we have detected GGA [XI] in human semen (unpublished; YT and YS).

In addition, mice bred and lactated by GGA supplementation showed increased expression of BDNF around the hippocampal dentate gyrus of mice at 1 week of age (40), suggesting that GGA supplementation also acts on brain development in the perinatal mice. Treatment of SH-SY5Y cells, a cell line derived from human neuroblastoma, with GGA [XI] results in morphological neuronal-like changes, including longer neurites and increased contact points per cell (35, 95), which is compatible with the results of the in vivo experiment. It is also worth mentioning the biological effects of GGA [XI] on bone metabolism. Namely, GGA [XI] induces osteoblast differentiation and inhibits osteoclast formation in vitro; GGA [XI] increases femur bone mineral density in SAM-P6 mice in vivo (96). The differentiation-inducing effect of GGA [XI] on neuroblastoma and its effect on bone metabolism may be due to the retinoid action of GGA [XI]. Because ATRA [X] is also known to have similar effects on neuronal differentiation of neuroblastoma (97) and osteogenesis (98).

Two pharmacological actions of GGA [XI] have also been observed: one is the inhibition of human immunodeficiency virus type-1 (HIV-1) infection. GGA [XI] inhibited HIV-1 infection of host cells; GGA is thought to inhibit HIV-1 entry into host cells by suppressing the cell surface expression of the chemokine receptor, CXCR4 (99). The other is the inhibition of lysine demethylase 1 (LSD1 or KDM1A), which belongs to the family of FAD-dependent amine oxidases that include MAOB. GGA [XI] and several of its dihydro derivatives inhibited active recombinant human LSD1 (100). Furthermore, GGA treatment of HuH-7 cells resulted in rapid migration of nuclear-localized LSD1 to the cytoplasm (101), indicating that GGA [XI] may have epigenetic effects on cells.

## Perspectives on GGA Research

### Further metabolites of GGA and their biological activities

We have described that GGA [XI] is produced as a metabolite of MVA in mammalian organs and cells, including humans, bovines, rats, and mice, and also even in parasites. GGA [XI] is further metabolized intracellularly to 2,3-dihydroGGA [XIII] in HuH-7 cells, in rat thymocytes (102), and in Wistar rat tissues, 2,3-dihydroGGA [XIII] has been detected several to more than a hundredfold (5-fold in the liver, 106-fold in the epididymis, and 141-fold in the thymus) higher than GGA [XI] (39). Foster *et al.* detected GGA [XI] and 2,3-dihydroGGA [XIII] in lipid extracts of *Schistosoma mansoni* after saponification of the triacylglycerol fraction, indicating that it may be a storage form of GGA [XI] and 2,3-dihydroGGA [XIII] (21). In our laboratory, we have observed that hydrolysis of the phospholipid fraction of rat liver and HuH-7 cells, especially the cardiolipin fraction, liberated GGA [XI] and 2,3-dihydroGGA [XIII] but could not detect them in the neutral lipid fraction (unpublished; YT and YS).

In addition, there are also differences in biological activities, such as the lipid droplet-inducing activity of 2,3-dihydroGGA [XIII] on cultured cells, which is not observed at all with GGA [XI] (103). Identifying GGA [XI] and 2,3-dihydroGGA [XIII] and their further metabolites, as well as confirming and analyzing their biological activities, are issues to be explored in the future.

### Essential role(s) of GGA in the cell

It was indeed surprising that even in KO cells of the *MAOB* gene, which encodes the enzyme responsible for GGA biosynthesis, the intracellular levels of GGA [XI] were maintained by induction of the backup enzyme CYP3A4. How does a certain level of intracellular GGA [XI] contribute to cell survival? Why should intracellular GGA [XI] not be reduced or lost in *MAOB*-KO cells? These points still need to be resolved.

### GGA as a primordial form of retinoic acid

We started with the study of the antitumor activity of retinoids, which led us to focus on the acyclic retinoid GGA [XI] as an endogenous metabolite of MVA. GGA [XI] is found in many eukaryotes, both plants and animals, from parasites deficient in steroid synthesis to mammals such as mice, rats, bovines, and humans. Needless to say, retinoids are essential nutrients and vitamin A that cannot be de novo biosynthesized in the animal kingdom, and their roles in ontogeny, cell differentiation, reproduction, and the nervous system are well known, making them essential for the survival of individual animals. This is a bit of a leap, but since GGA binds to CRABP and exhibits ligand activity for RAR and RXR, we hypothesize that GGA might have emerged as the primordial molecule of retinoic acid in eukaryotes, acting now as an essential signal transduction molecule for cell survival.

## Conflict of Interest

The author declares that they have no conflicts of interest with the contents of this article.
